# Complementary medicine use among Moroccan patients with cancer: A descriptive study

**Published:** 2011-11-11

**Authors:** Sami Aziz Brahmi, Fatema Zahra El M'rabet, Zineb Benbrahim, Yusra Akesbi, Berraho Amine, Chakib Nejjari, Omar El Mesbahi

**Affiliations:** 1 Medical Oncology Unit, Hassan II University Hospital, Fez, Morocco; 2 Department of Epidemiology, Hassan II University Hospital, Fez, Morocco

**Keywords:** Complementary medicine, cancer, alternative medicine, Morocco

## Abstract

**Background:**

Complementary and alternative medicine (CAM) is a group of diverse medical and health care systems, practices, and products that are not generally considered part of conventional medicine. As cancer incidence rates and survival time increase, use of CAM will likely increase. However, little is known about the use of CAM in cancer patients, specifically in emerging countries.

**Methods:**

We conducted a study in the medical oncology department at the University Hospital of Fez on the use of complementary medicine among cancer patient. The aims of this study were to estimate and describe the reasons of use of complementary medicine (CM) in patients with a cancer treated in a Moroccan oncology department. A specially designed questionnaire was completed for patient during treatment or follow-up in the oncology department after formal consent was obtained. It was a descriptive study among 100 patients over a period of 6 months between September 2008 and February 2009.

**Results:**

A total of 100 patients participated in the study, 46 of them were identified as users of complementary medicine. The most substances used were plants 24%, pure honey 13% and water of Zem Zem (holy water from Mecca) 11%. Concerning techniques, religious practices 37%, special diets 22% and recourse to traditional healers 11% were most commonly used. No specific user profile was observed depending of different sociodemograhics and clinical parameters. The majority of the users of complementary medicine were not revealing their habits to their oncologist because the question was not raised in consultation.

**Conclusion:**

It seems that medical doctors should ask patients about their use of complementary medicine when they obtain medical history and they need to know more about complementary medicine to offer better consultation. Complementary medicine must benefit, as well as conventional medicine, from scientific studies to evaluate potential benefits, toxicity and interactions with the conventional treatment to enable the oncologist better inform his patients.

## Background

Complementary and alternative medicine (CAM) is a group of diverse medical and health care systems, practices, and products that are not generally considered part of conventional medicine [1]. The last decade has seen an increase of use of complementary and alternative medicine in the world especially in patients suffering from a chronic disease such as cancer. However, little is known about the use of CAM in cancer patients specifically. This is especially true among Africa and Arabic countries, as the bulk of the literature comes from the United States of America.

In Morocco, the terms "complementary medicine" or "alternative medicine" are used inter-changeably with traditional medicine. They refer to a broad set of health care practices that are not part of that country′s own tradition and are not integrated into the dominant health care system. Information on CAM use in comprehensive cancer centers is limited and outdated. Although we acknowledge that use of these therapies is common, information on contemporary use, attitudes, and beliefs is needed for clinicians, program planners, and patient educators who must respond to the growing interest among patients, particularly in the comprehensive cancer centers. The purpose of this study was to examine CAM use in a more current, representative sample of cancer outpatients.

We conducted a study in the medical oncology department at the University Hospital of Fez on the use of complementary medicine among cancer patients.

## Methods

The aims of this study were to estimate and describe the reasons for use of complementary medicine (CM) and to elaborate the type, the perception, the source and the disclosure of CM use in patients with a cancer treated in a Moroccan oncology department. A specially designed questionnaire was completed for patients during treatment or follow-up in the oncology department after formal consent was obtained. It was a descriptive study of 100 patients over a period of 6 months between September 2008 and February 2009.

### The questionnaire

The questionnaire used was based on the one developed by Trager Maury and al [2]. However, the questionnaire was modified for the purposes of the present study. The questionnaire was administered by a doctor. There were 17 items in total. These included demographic data (age, gender, occupation, education, household income, and marital status), clinical data (site of primary cancer, standard treatments received previously and current standard treatment) and questions about CM use.

If patients reported no past or current use of CM after completing the sociodemographic and clinical section of the questionnaire, they were asked to choose an answer from a list of possible reasons for not using CM. After that, patients were thanked for their contribution and the completion of the questionnaire was stopped at that stage. If patients reported past or current use of CM we continued administering the questionnaire. The rest of the questions asked were:

Which CM therapy patients used before the diagnosis of cancer, since the diagnosis of cancer or currently, this was done from a list containing 4 groups: herbs, vitamins, dietary regimens, spiritual therapies; reasons for using CM therapies; sources of information about CM therapies; satisfaction and perceived effectiveness (on a 0–10 scale with higher scores indicating higher levels of satisfaction or perceived effectiveness); expenditure on CM.

### Data analysis

Data were analyzed using the Statistical Package for Social Sciences (SPSS) program. Descriptive statistics 13.0 were calculated with all variables to summarize the data. Differences in sociodemographic and clinical characteristics between CM users and non-users were assessed using statistical tests, comparisons by X^2^ analyses were used to assess predictors of CM use.

## Results

A total of 100 patients participated in the study. Females represented 66% of the group. Their mean age was 48 years (range 26-70; standard deviation (SD) 13). Two thirds of patients were illiterate. Regarding the professional categories, one third of the sample is composed of housewives and fifth by the workers and farmers. Most (72%) were earning less than 1 500 Moroccan dirhams monthly (approximately 150 Euro). The 2 most common cancers represented were breast cancer with 44% and colon cancer with 18%. The distribution of other cancers was as follows: ovarian cancer: 8%, stomach cancer: 7%, soft tissue cancer 4%, rectum: 2%, lung cancer: 9%, pancreas cancer: 1%, nasopharyngeal cancer: 4%. For tumor stage, almost half of patients had locally advanced disease (46%), localized and metastatic disease frequency were similar: 27%. The treatment used for cancer was both surgery and chemotherapy for 57% of patients and chemotherapy alone for 30% of patients. Treatment was ongoing for 80% of cases.

Among the 100 patients included in the study 46 were identified as users of complementary medicine. Regarding the initial setting of the CM, 48% (22 patients out of 46) used the CM before the diagnosis of disease and 39% after diagnosis of their disease. It may be noted that these 39% could be divided into 3 subgroups: 21% have used the CM when they were under treatment (10 patients), 10% (5 patients) started the CM cancer diagnosis and 8% (3 patients) started when they knew they were going to have chemotherapy or radiotherapy. Of the 46 patients, 6 had regular recourse to the CM if they developed a health problem.

**Figure 1:**
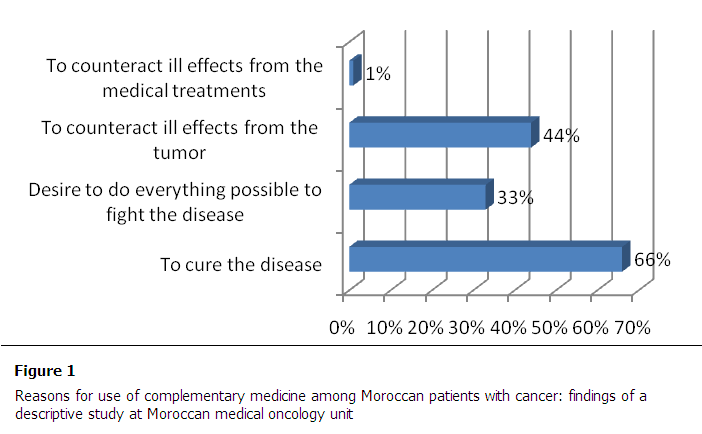
Reasons for use of complementary medicine among Moroccan patients with cancer: findings of a descriptive study at Moroccan medical oncology unit

To the question "why do you use the CM to treat your disease? The 2 main reasons were to cure disease and relieve symptoms associated with it. The distribution of different reasons is given in Figure 1.

**Figure 2:**
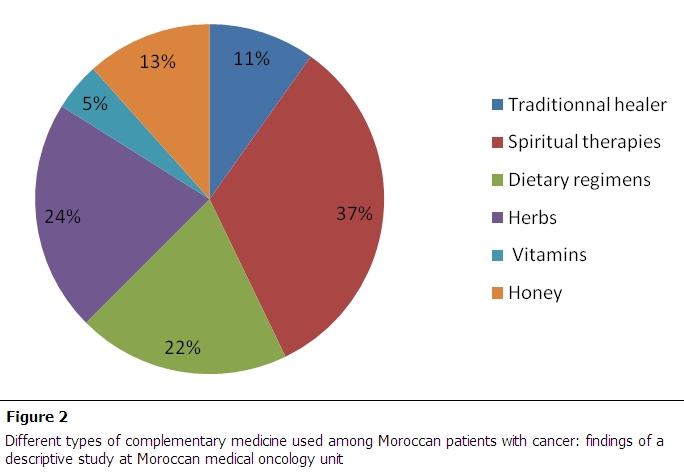
different types of complementary medicine used among Moroccan patients with cancer: findings of a descriptive study at Moroccan medical oncology unit

Among the 46 users of complementary medicine, 48% use substances and 20% techniques. The most substances used were plants 24%, pure honey 13% and water of Zem Zem (holy water from Mecca) 11%. Patients procured medicinal plants from an herbalist or traditional healer. The most reported plant was *Aristolochia longa*, braztam in Moraccan dialect. Other plants have been cited: *Euphorbia officinalis*, *Lavandula stoechas*, *Scirpus holoscenus*, *Juniperus thurifora*. Concerning techniques, religious practices (37%), special diets (22%) and recourse to traditional healers (11%) were most commonly used. Figure 2 summarizes the different medicines used. Also 21% of patients admitted that they have intensified their religious practices after diagnosis of cancer.

The main information source of complementary medicine was patient's family and friends for 65% of patients (30 patients out of 46). Other sources were the traditional healer for 17% (8 patients) and the media for 8% (4 patients). Regarding the monthly budget devoted to complementary medicine, the average was 26 dirhams, with extremes from 0 to 300 dirhams (0 to 30 Euros).

Users of complementary medicine and non-users of complementary medicine do not differ significantly by sex (p = 0.29). The 2 groups had roughly the same average age, with respectively 49.1 and 46.6 years for users and non users. No specific user's profile was observed depending of different sociodemograhics and clinical parameters. Patients tended to be satisfied with the use of CM and they also felt the particular therapy used was effective. The mean satisfaction score was 6.5 where a score of 10 indicated the highest level of satisfaction.

The answers to the question "Do you think that complementary medicine can have side effects? "Were classified into 3 categories: yes, no and I do not know. The principle was the same for the perception of interactions of complementary medicine with conventional treatment. The answers to this question showed that 50% of users believed that complementary medicine can give side effects. In contrast, 74% of non-users did not know answer to this question. Users of CM do not differ from non-users regarding the perception of interactions complementary medicine / conventional treatment. Forty three percent of users believed that there were no interactions (20 out of 46 users). Forty six percent of non-users did not know whether there was an interaction between the 2 treatments.

**Figure 3:**
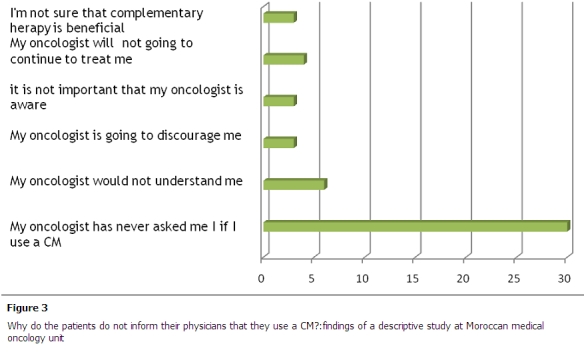
Why do the patients do not inform their physicians that they use a CM?:findings of a descriptive study at Moroccan medical oncology unit

The majority of the users of complementary medicine were not revealing their habits to their oncologist because the question was not raised in consultation (Figure 3).

Fifty four percent of patients did not use complementary medicine. The two most cited reasons were that patients were skeptical about the effectiveness of such medicine or were discouraged by their families and friends.

## Discussion

The study population was dependent on one service from a single hospital; some cancers were poorly represented such as cervical cancer and cancers of the head and neck. This explains the presence of a large number of women in our study population. The generalization of these results to the population of cancer patients is not possible.

The questionnaire was administered by 3 investigators; because of the high rate of illiteracy it was impossible to give the questionnaire to patients to complete themselves. This had both advantages and disadvantages. Firstly the investigator assisted the patient in understanding the issues and clinical information was collected accurately but on the other hand, it could discourage freely disclose of use of complementary medicine.

Approximately half (46%) of patients in this study used complementary medicine. This coincided with the main proportion of use found in the literature. A European study in 2004 among 956 patients showed proportions of use of complementary and alternative medicines ranging from 15 to 75% with an average of 35.9% [3]. A meta-analysis of 26 studies from 13 countries showed a ratio of 7 to 64% of users of complementary and alternative medicine with an average of 31.4% [4]. However, this prevalence depends on 3 criteria: the definition of complementary and alternative medicine, medicine included in alternative medicine and complementary and type of population studied. For example the study conducted by Trager-Maury et al found a prevalence of 34% of users, but the spiritual and religious practices were not included as complementary medicine [2]. Similarly, the study conducted by Sollner et al found 24% of users, in an Austrian population with cancer.

The results of this study showed no difference between users and non users of complementary medicine regarding age, sex, profession, type of cancer, treatment and stage of disease. Indeed, several studies have attempted to determine a profile of users of CM. According to a recent systematic review of 43 studies, the user profile is as follows: age between 30 and 50, female, with significant income, high education level, and a tumor at an advanced stage [5]. However, it should be noted that the studies in this review didn't include African populations, and Arabic countries.

In our study the main source of information on complementary medicine was family and friends. Another important source is found in the literature. Although the Internet is largely used in Morocco as an information source, it is not a major one for patients according to our study.

The most motioned types of complementary medicine used in cancer in the literature are vitamins, minerals and plants [7,8]. In our study, the most substances represented were plants and pure honey. Religious practices, special diets, and the recourse to healers were the most commonly used techniques. Some users of complementary medicine used them prior to their illness and continued when they were suffering from cancer. This use was part of a family habit, and it was most often use of substances such as plants. Also, certain substances in our study were reported by a qualitative and quantitative study on the state of knowledge, perceptions and attitudes towards cancer in Morocco conducted by Lalla Salma Association against cancer [9]. According to this study, plants or ingredients commonly used to treat cancer are often aggressive or have toxic effects. Particular attention should be paid to Aristolochia (*Aristolochia Longa*) by far the most important plant used, called in Arabic Chajarat Rust, and Moroccan dialect: berztem or Gettat el hmir lberri [10]. In fact, Aristolochia is very toxic and irritating; it could cause serious eye diseases, respiratory and digestive tracts irritation [10]. It is also a carcinogen and teratogen [10]. A proportion of 21% of patients with CM user used this therapy when they were undergoing treatment. It is important to ask the patient about complementary medicine as often as possible during the consultation prior chemotherapy.

In our study, the main reason for use complementary medicine was to cure the disease. The reasons given in other studies conducted in Europe and the United States were to improve the quality of life including the side effects and immune system stimulation [5]. For the efficiency perception of conventional medicine and complementary medicine users and non-users patients felt that conventional medicine is more effective than complementary medicine.

The majority of the users of complementary medicine were not revealing their habits to their oncologist because the question was not raised in consultation. This coincides with the literature which found the same reason [11]. But this discussion is important because a significant proportion of users ignore the risk of side effects or interactions of these therapies with conventional medicine.

## Conclusion

It seems that medical doctors should ask patients about their use of complementary medicine when they obtain medical history and they need to know more about complementary medicine to offer better consultation. Complementary medicine must benefit, as well as conventional medicine, from scientific studies to evaluate potential benefits, toxicity and interactions with the conventional treatment to enable the oncologist better informing his patients. From our point of view larger studies are needed. Efforts should be made to better inform patients, and incorporate some aspects of the MC in the public health system.
